# Heat Flux Analysis and Assessment of Drying Kinetics during Lyophilization of Fruits in a Pilot-Scale Freeze Dryer

**DOI:** 10.3390/foods12183399

**Published:** 2023-09-11

**Authors:** Ivan Sedmak, Matic Može, Gorazd Kambič, Iztok Golobič

**Affiliations:** 1 Laboratory for Thermal Technology (LTT), Faculty of Mechanical Engineering, University of Ljubljana, 1000 Ljubljana, Slovenia; matic.moze@fs.uni-lj.si (M.M.); iztok.golobic@fs.uni-lj.si (I.G.); 2 Kambic Laboratory Equipment, 8333 Semič, Slovenia

**Keywords:** drying kinetics, fruit drying, heat flux measurements, lyophilization, sublimation endpoint, temperature distribution

## Abstract

Vacuum freeze-drying as a process for achieving high product quality has attracted increasing attention in the last decade. Particularly in the pharmaceutical field and food processing industries, lyophilization can produce high-quality products compared to samples dried by conventional methods. Despite its benefits, lyophilization is a time-consuming and costly process that requires optimization of a number of process parameters, including shelf temperature, chamber pressure, freezing rate, and process time. This paper reports on the implementation of heat flux measurements that allow noninvasive real-time determination of the endpoint of the primary drying stage as an essential parameter for the effective optimization of the overall drying time. Quantitative analysis of the drying kinetics of five fruits (kiwifruit, avocado, Asian pear, persimmon, and passion fruit) was assessed by comparing the heat flux and temperature profiles of samples during the lyophilization process. For a 24 h lyophilization cycle, average heat fluxes in the primary drying phase ranged from 250 to 570 W/m^2^. A significant correlation was found between the temperature and heat flux distributions at the estimated endpoint of the sublimation process and the corresponding transition into the secondary drying stage. Furthermore, good agreement was also found for the freezing phase. The use of real-time heat flux measurements proved to be a cost-effective experimental method to better understand the process variables in order to reduce the lyophilization cycle time and overall energy consumption.

## 1. Introduction

Vacuum freeze-drying is widely recognized as the gentlest dehydration process among various drying technologies. In the pharmaceutical industry, for example, it allows the preservation of the structure and viability of many types of microorganism strains and biological substances [1,2]. As a very effective low-temperature dehydration process, it is also recognized as one of the best methods for preservation of high-quality foods, especially those prone to thermal oxidation [3,4,5,6]. In principle, lyophilization is realized through three stages: (i) freezing stage, (ii) primary drying stage, and (iii) secondary drying stage. Starting with the freezing stage, the water content is (typically) allowed to freeze. At the end of this stage, the ambient pressure is reduced below the triple point pressure and the unbound water in the sample begins to sublimate. The sublimation process is further enhanced by heating the sample to a favorable temperature using a high-precision shelf temperature controller. Particular care must be taken to keep the sample temperature below its critical value (i.e., the glass transition temperature *T_g_* for amorphous and certain crystalline samples). Equally important is the need for precise control of the sample temperature to ensure the required structural integrity during the primary drying stage. When optimizing the drying process of the production batch, this can be challenging as a nonoptimized drying cycle combined with elevated shelf temperature can lead to the inevitable cake shrinkage or collapse and, thus, product degradation [7,8].

Until recently, most monitoring and evaluation studies of lyophilization processes were based on the analyses of temperature and pressure measurements. For example, a comparative Pirani pressure measurement and sample temperature measurement are often the preferred methods for determining the apparent endpoint of the sublimation process [9,10]. The approach is based on the principle of measuring the pressure difference between the capacitance manometer and the Pirani manometer. As the sublimation endpoint is reached, the pressure difference tends to decrease toward zero. Alternative approaches for assessing the end of primary drying are described in detail by Vollrath et al. [11]. Among these approaches, tunable diode laser absorption spectroscopy (TDLAS) and mass spectrometry can reliably detect the endpoint of primary drying but are limited to whole-batch determination. Furthermore, many of these approaches have been applied to sensitive pharmaceuticals and have yet to be fully realized, particularly in food processing applications [6].

As mentioned above, temperature sensors are essential for assessing the duration of the three main stages of lyophilization. This is particularly true for the primary drying stage, which is usually the longest stage and where most of the unbound water is removed. The most commonly used temperature sensors in lyophilization applications are thermoelectric and resistance sensors, although wireless temperature sensing is becoming increasingly popular in recent years, particularly in the pharmaceutical industry [12]. When monitoring the product temperature *T_p_* at the bottom of the test dish, flask, or vial, attention must be paid to changes that may affect the sublimation rate and the quality of the cake structure. This is important because an inappropriate placement and/or size of the temperature sensor can have a significant effect on lyophilization behavior, which in turn can lead to misinterpretation of the sublimation process and nonoptimized lyophilization cycles. The latter fact is particularly relevant when the volume of the sample is comparable to the volume of the sensor itself, as in the case of wireless sensors (e.g., temperature remote interrogation system, TEMPRIS). However, to fully understand the freeze-drying behavior of the product batch, invasive temperature measurements are currently unavoidable.

One of the most promising, cost-effective, and practical methods for determining the end of the primary drying is based on measuring the heat flux between the shelf and the dish/vial [11]. The use of the heat flux sensor to detect various thermal events during lyophilization of mannitol solutions was demonstrated by Juckers et al. [13]. Moino et al. [14] have pointed out that it is also possible to monitor the nucleation event and the subsequent growth of the ice crystals. Furthermore, this noninvasive method allows monitoring of several process parameters, such as the heat transfer coefficient (*K_v_*), mass flow rate, sample resistance (*Rp*), changes in heat flow direction, and process kinetics. Another advantage is the ability to provide real-time measurements regardless of the equipment used (e.g., laboratory freeze dryer, product freeze-drying system, etc.). Although there are certain limitations in terms of the potential restriction of heat transfer along the domains, the thinness of novel thin-film heat flux sensors offers relatively low thermal resistance and allows them to be placed in a particular area of interest. In addition, depending on the design, some types of heat flux sensors are implemented as passive sensor elements that do not require an external power supply. This is advantageous when investigating local heat transfer characteristics as there is no parasitic heat generation (i.e., Joule heating).

The aim of the present study is to investigate the correlation between heat flux and temperature distributions at the estimated endpoint of the sublimation process during lyophilization. Our approach to heat flux measurement is based on a single vial or localized assessment that can differentiate between edge and center vials. This differs from the established approach of measuring multiple vials [14,15]. The study discusses the potential use of the heat flux sensor alone for noninvasive detection of the nucleation event during the freezing phase by monitoring the heat flow rate and the readings of a thermocouple embedded in the sensor. Experimental measurements were performed using a thin-film heat flux sensor with an integrated thermocouple (TC) positioned between the shelf surface and the glass vial, while a reference thermocouple was placed inside the vial itself. The drying kinetics of different fruits were analyzed to determine the effect on the drying rate and primary drying time and the influence of moisture content on the energy flow.

## 2. Materials and Methods

### 2.1. Fruit Preparation and Packaging

In this study, the fruit samples to be analyzed were purchased from a local grocery store in the city of Ljubljana (Slovenia) and prepared on the same day. The characteristics of the fruits analyzed are presented in Table 1.

**Figure 1:**
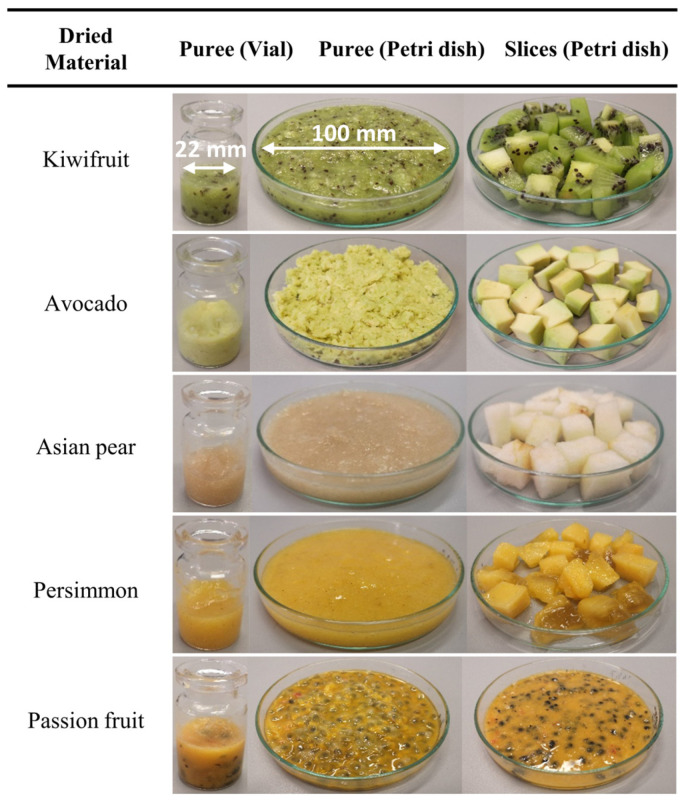
Preparation of fruit samples to be freeze dried.

The Shimadzu AX200 analytical balance was used to accurately weigh the samples during preparation. In order to make the samples more homogeneous and to facilitate the freeze-drying process, all thick-skinned fruits were peeled, sliced, and pureed using a commercial kitchen blender for 20 s. It was assumed that foaming, an unavoidable side effect of the mixing process, could favorably promote mass transfer and consequently shorten the drying time because of the increased gas–liquid interfacial area. Approximately 4 g of fruit puree was filled into a glass vial, while the remainder was poured into a 100 mm diameter round Petri dish to form a sample layer of about 8 mm. The latter was used as a reference to assess the total amount of moisture removed during the particular lyophilization cycle. As shown in Figure 1, to verify compliance with the above moisture removal efficiency, we also added another Petri dish filled with small fruit slices. SCHOTT 6R ISO (standard) glass vials with an overflow capacity of 10 mL and a diameter of 22 mm were used for heat flux measurements of the fruit puree.

### 2.2. Freeze-Drying Protocols

Freeze-drying of the fruits was carried out in a pilot-scale freeze dryer (Lio-10P; 0.1 m^2^; Compact stand-alone unit, Kambič d.o.o., Slovenia). As summarized in Table 2, several freezing protocols were used to evaluate the influence of different process parameters on the endpoint of the sublimation process.

Individual fruits were prepared in two Petri dishes and in one vial and then placed in the freeze dryer at approximately 25 °C. The shelf temperature was ramped to the final freezing temperature of −35 °C (predefined ramp rate 100 °C/h). The samples were then allowed to equilibrate for 2 to 3 h to ensure the complete solidification of all freezable components. It should be noted that rapid freezing generally favors the formation of small intracellular ice crystals, thus slowing down the primary drying. On the other hand, the experimental observations have shown that the presence of small crystals preserves the cellular structure of the food and also intensifies secondary drying [6]. Therefore, due to time constraints and the relatively short secondary phase, the rapid ramp rate was chosen deliberately.

After the completion of the freezing step, the pressure was reduced to either 0.14 or 1.00 mbar for primary drying. Preliminary experiments were carried out to assess the stability and quality of the samples at higher drying temperatures (i.e., under aggressive lyophilization conditions), which were necessary to reduce the total drying time to less than 24 h. Accordingly, the relationship between different process parameters was investigated to determine the optimum shelf temperature to be around 5 °C. It is noteworthy that although the adjusted shelf temperature was above the freezing point, the sample temperature during the primary drying always remained below the freezing point.

### 2.3. Heat Flux Determination

**Figure 2:**
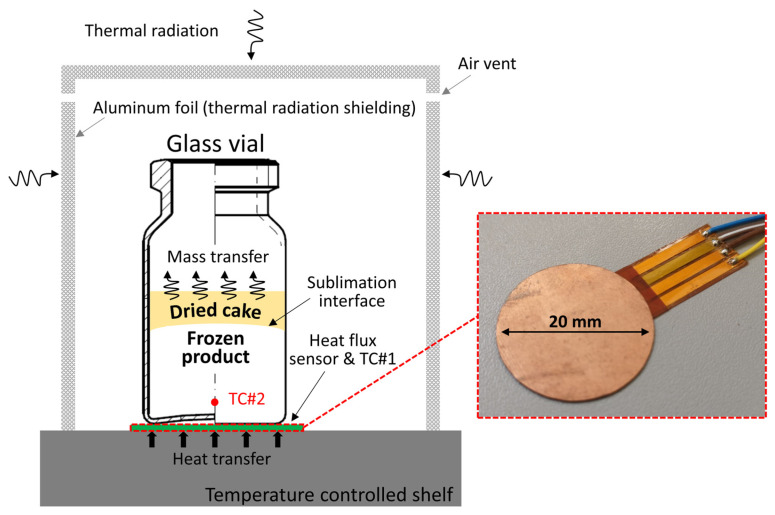
Schematic representation of the vial and the processes involved in freeze-drying (**left**) and a photograph of the thin-film heat flux sensor (**right**).

A heat flux sensor (Captec Entreprise, Lille, France) was used to measure the conductive heat transfer between the tempered shelf and the glass vial. The vial was precisely placed on the sensing area to obtain reliable data. The heat flux sensor itself consisted of several thin-film layers with a combined thickness of 0.36 mm and a diameter of 20 mm, providing a sensing area of 315 mm^2^. The sensitivity of the sensor was 3.7 µV/W/m^2^, while the response time was found to be around 0.3 s. The sensor itself contained an embedded TC#1 thermocouple (T-type; copper/constantan), which was used for the shelf temperature measurements. As shown in Figure 2, a custom-designed reference sensor TC#2 (T-type; Labfacility Limited, South Yorkshire, UK) with a spherical junction diameter of 0.41 mm was placed at the center bottom of the vial to invasively measure the sample temperature during the lyophilization process. Its small size allowed reliable tracking of the temperature transition from steady-state sublimation to equilibration conditions at shelf temperature. The Agilent 34970A (Agilent Technologies Inc., Santa Clara, California, USA) data acquisition unit was used for both heat flux and temperature measurements. Measurements were collected at a sampling interval of 1 min. Thin aluminum foil was wrapped around the vial/heat flux sensor assembly to minimize radiative heat exchange with chamber walls. In addition, small vents were made in the aluminum wall surfaces to allow water vapor to escape during the lyophilization process.

### 2.4. Determination of Vial Heat Transfer Coefficient

The pressure difference between the solvent saturation pressure at the sublimation interface and the solvent partial vapor pressure above the sample surface is the main driving force of the sublimation process. Therefore, the heat transferred to the sample directly affects the solvent saturation pressure and the magnitude of the sublimation rate. The heat flux applied to the bottom of the vial can be expressed as:

(1) $$\dot{Q}=K_{v}A_{v}\left(T_{sh}-T_{v}\right)$$

where $\dot{Q}$ is the heat transfer rate, *K_v_* is the vial heat transfer coefficient between the shelf and the bottom of the vial in the primary drying stage, *A_v_* is the outer vial bottom area, *T_sh_* is the shelf temperature, and *T_v_* is the sample temperature at the bottom of the vial.

It should be emphasized that there are three major heat transfer contributions to *K_v_*: (i) thermal conduction between the shelf and the vial bottom, (ii) thermal conduction through the gap between the curved vial bottom and the shelf, (iii) and thermal radiation between the vial and the shelf. In our case, however, the heat transfer coefficient was assessed by monitoring the heat flux and, in addition, the temperatures at bottom of the vial and the temperature of the shelf. Thus, in the quasi-stationary phase of primary drying, the *K_v_* was calculated by using the following equation [11]:

(2) $$K_{v}=\frac{\dot{q}}{\left(T_{sh}-T_{v}\right)}$$

where heat flux $\dot{q}$ and *T_sh_* are measured by the heat flux sensor.

## 3. Results and Discussion

### 3.1. Measurements of a Nucleation Event during the Freezing Phase

When monitoring the nucleation event during the freezing phase, particular attention should be paid to the pattern of the temperature variations. For example, the irregular temperature pattern is usually a manifestation of a stochastic nucleation event starting from the bottom of the dish/vial and progressing upward. This uncontrolled nucleation behavior can significantly affect the ice crystal morphology and pore structure, which in turn can have a negative impact on the sample quality, uniformity, and appearance. Another problem that cannot be avoided in the freezing process is the use of temperature sensors to measure the sample temperature. Their “invasive” effect on the sample morphology can be significant: (i) problems can arise with regard to a potential thermal bridge, which can promote heat transfer in the lyophilized sample, and (ii) with regard to an additional source of nucleation. 

**Figure 3:**
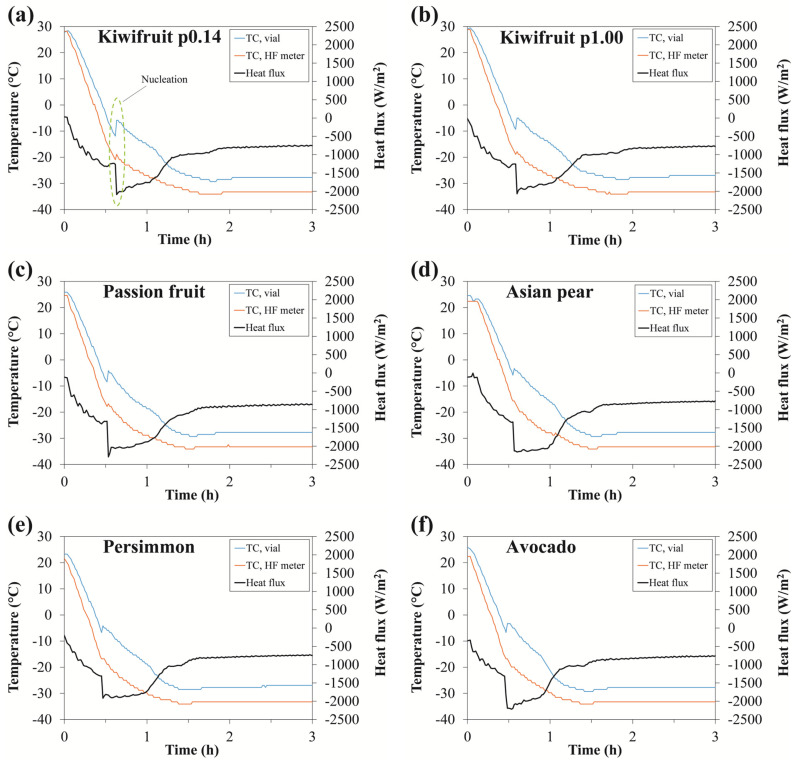
Comparison of the heat flux and temperature profiles during the freezing phase for different types of fruit. (**a**) Kiwifruit at 0.14 mbar; (**b**) Kiwifruit at 1.00 mbar; (**c**) Passion fruit; (**d**) Asian pear; (**e**) Persimmon; (**f**) Avocado.

Therefore, the idea behind our approach was to compare the ability of the heat flux sensor to noninvasively detect the onset of ice crystallization with the temperature response of the sensor-embedded TC#1 and reference TC#2 thermocouple. Figure 3 shows the heat flux and temperature profiles during the freezing phase of different types of fruit. In our case, due to the limitations of the apparatus, only an uncontrolled (random) nucleation process was possible. This was the main reason why the variable state between the equilibrium freezing temperature and the ice nucleation temperature (the so-called “degree of supercooling”) was not detected, as it could potentially be in the case of controlled nucleation. As can be clearly seen from the figure, a sharp decrease in heat flux was detected at around −12 °C, thus indicating the supercooled state and the spontaneous formation of initial ice nuclei. In all cases, the nucleation event occurred less than one hour after the start of the lyophilization cycle, which was further confirmed by the temperature response of the thermocouple placed at the center bottom of the vial. As indicated by the green circle in Figure 3a, a sharp peak of 8 °C was measured at the point of the ice nucleation in the vial. No additional humps in the temperature profiles were observed here, as is usually the case with surrounding neighboring vials [16]. Thereafter, the temperature tended to rise toward the equilibrium freezing point of water but never reached this level because of the rapid ramp-down rate of 100 °C/h. However, this sharp rise in temperature indicated that the temperature measurements alone might be sufficient to detect the nucleation event. In contrast, the thermocouple embedded in the heat flux sensor did not show a significant temperature response. This is namely due to the considerable distance between the location of the advancing solidification (freezing) front within the vial and the embedded thermocouple, making these measurements less informative. Nevertheless, there is some indication of the temperature rise from the embedded thermocouple in cases a-c of Figure 3. It is reasonable to assume that the increase would be even more pronounced if the filling mass were larger.

Finally, it should also be noted that in order to ensure reliable and representative temperature measurements of the nucleation event, temperature sensors with small dimensions should be selected. Furthermore, as the temperature is measured invasively within the sample, a large temperature sensor can also seriously affect the nucleation process itself [17,18].

### 3.2. Measurement of Heat Transfer Coefficients

The heat transfer coefficient between the shelf and bottom of the vial was determined by measuring the heat flux through a single vial at different operating conditions. Two different freezing protocols were used to assess the effect of chamber pressure on the *K_v_* coefficient. Table 3 shows the *K_v_* values obtained for the freeze-drying of the kiwifruit at chamber pressures of 0.14 mbar and 1.00 mbar.

**Figure 4:**
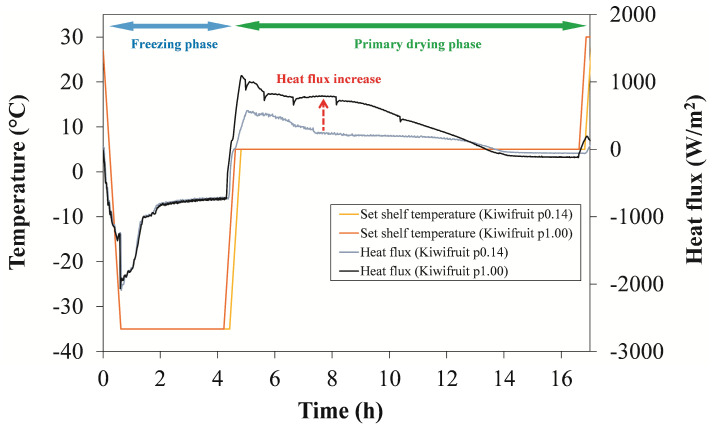
Heat flux profile over time for drying of the kiwifruit at a chamber pressure of 0.14 mbar and at 1.00 mbar.

The presented *K_v_* values were determined according to Equation (2) during the 4 h of quasi-steady-state primary drying during which the heat flux remained at a relatively constant level. As depicted in Figure 4, a large increase in heat flux was observed at a chamber pressure of 1.00 mbar. As expected, the increased chamber pressure resulted in a more intense heat transfer between the shelf and the vial because of the increased thermal conductivity of the gas trapped in the concave bottom of the vial, as the conductivity of water vapor is highly pressure dependent [19]. Such differences in heat flux values could also be attributed to the fact that there was no shielding from the surrounding dummy vials. This further suggests that the overall heat transfer characteristics of the individual vial can be significantly different from that of a vial shielded by surrounding vials [20].

The determination of *K_V_* by the heat flux sensor may differ from that obtained by gravimetric analysis. This is due to the fact that, compared to gravimetric measurements, we can only measure conductive heat transfer and no other mechanisms such as radiation. On the other hand, the main drawback of gravimetric analysis is the necessary disturbance of the heat flow associated with the protocols of lifting and weighing the vial.

In Figure 4, we would like to highlight another aspect of these measurements relating to freezing time. The actual freezing time at the higher chamber pressure is longer than the set freezing time. This is due to the way this freeze dryer works, as the transition to the next stage (primary phase) is only possible once the set chamber pressure has been reached. With a higher chamber pressure setpoint, this transition was slower, although the freezing time setpoint was shorter (Table 2). This is presumably due to the difficulty in achieving the desired chamber pressure caused by the intense sublimation involved.

### 3.3. Measurements of the End of the Primary Phase

**Figure 5:**
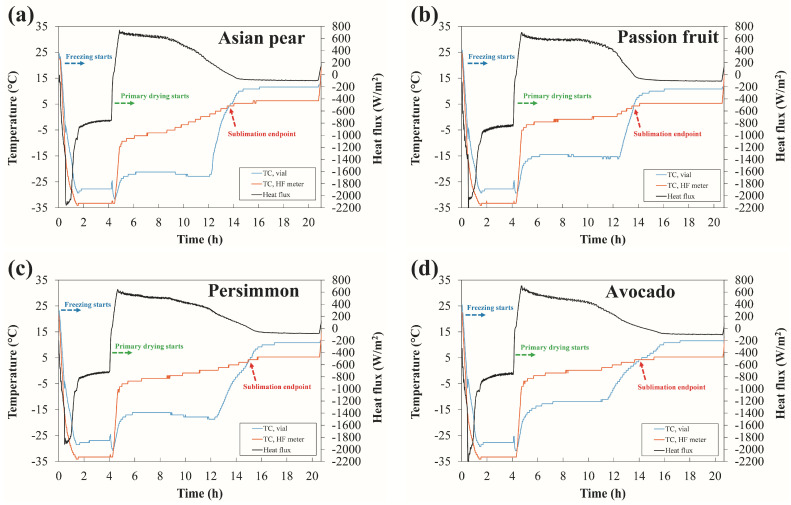
Comparison of the kinetics of primary drying for different types of fruit as determined by the thermocouple in the vial and by the heat flux sensor. (**a**) Asian pear; (**b**) Passion fruit; (**c**) Persimmon; (**d**) Avocado.

The main purpose of the proposed experimental approach was to assess the end of the primary drying phase of the fruit by comparing the measured heat flux and temperature distributions. In fact, the heat flux measurements provided further information confirming the already known fact that the sample temperature measurements alone can potentially detect the endpoint in the lyophilization process. As can be seen from Figure 5, the drying kinetics of selected fruits were successfully obtained within predetermined short lyophilization cycles. As expected, the highest peak in heat flux was observed in the initial time interval between 4 and 6 h, corresponding to the maximum mass flow rate. This was followed by a steady decrease in the mass flow rate until stable operation of the primary drying was established. It is worth noting that this steady decrease was associated with the increased resistance to vapor flow within the partially dried cake.

The transition from the primary drying phase toward the secondary drying phase was characterized by an increase in the temperature at the vial bottom. The heat flux, on the other hand, decreased rapidly toward zero, indicating the apparent end of the sublimation process. Before proceeding with the analysis, it is important to note that at the end of the primary drying phase, some moisture may still be present in the sample. Determination of the moisture content was not within the scope of this study; therefore, the residual moisture content, which can typically be determined by Karl Fisher (KF) titration or thermogravimetric analysis, was not analyzed. In this respect, the extent of potential shrinkage of the samples tested because of the presence of excess moisture was not assessed.

One focus of the study was to determine a rapid-drying-time protocol to provide sufficient datasets for further comparison and endpoint determination. As mentioned above, the end of the primary drying phase was associated with an increase in sample temperature. It should be noted that an increase in sample temperature may not only be due to the end of sublimation but also to a premature transition to secondary drying. It is therefore recommended to prolong the primary drying sufficiently and/or to check the end of sublimation by parallel measurements with the reference methods mentioned above. In our case, however, this was verified using the heat flux sensor. The heat flux profiles shown in Figure 5 indicate that the endpoint of sublimation was reached when the heat flux passed zero value and the sharp rise in temperature occurred. Furthermore, both temperature profiles made the sudden rise and then crossed each other at the exact moment when the heat flux reached zero value. This further indicates that the sublimation front had already passed the location of the thermocouple junction in the vial, while a narrow layer of the frozen region remained. After this event, the sample temperature gradually approached the steady-state conditions at about 10 °C, thus indicating the completion of primary drying. Due to the lack of shielding from the surrounding dummy vials, the sample temperature was higher than the set shelf temperature. It can be assumed that this effect is more pronounced in smaller freeze dryers such as the one used in this study [19]. This is partly due to differences in air chamber temperature and scattered radiation from the chamber walls that has escaped through gaps in the aluminum shielding foil.

It should be pointed out that a comparative pressure measurement using the Pirani sensor as a method of determining the endpoint of the sublimation process may not always be the best choice. The reason for this is the potential leakage and/or residual moisture entrapments within the freeze-dryer chamber, its tubes, and condenser cavity. This, in turn, can affect the readings of the Pirani sensor and can lead to a false interpretation of the end of the sublimation process. In particular, the condenser cavity could be a potential issue, misleading the Pirani pressure measurement, as it is known that its temperature is usually not uniform and that the vapor pressure of ice can vary significantly because of temperature differences. For this reason, and for the sake of simplicity, we recommend the use of the heat flux sensor as the main tool for the optimization of the primary drying phase.

### 3.4. Measurements of the End of the Secondary Phase

**Figure 6:**
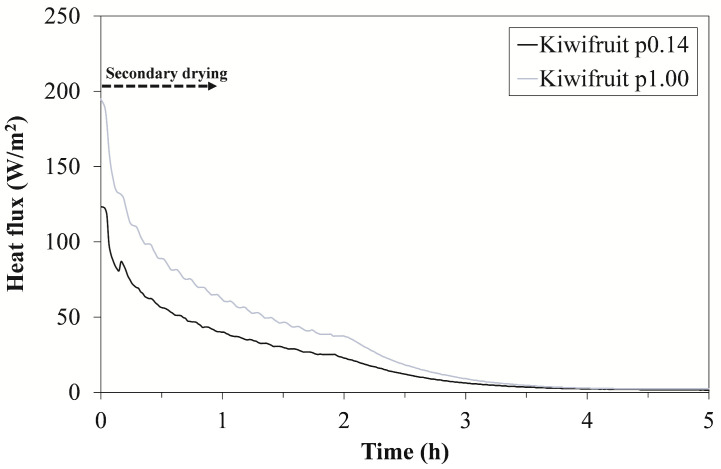
Heat flux profiles for kiwifruit during secondary drying at a chamber pressure of 0.14 mbar and at 1.00 mbar.

Secondary drying takes place at higher shelf temperatures, which are required to achieve a sufficient level of desorption efficiency. Figure 6 shows a gradual decrease in heat flux during the secondary drying phase of the kiwifruit. The maximum heat flux values for the two protocols used (Table 2) varied considerably, ranging from 125 to 190 W/m^2^ before dropping rapidly to zero. Both trends suggest that the thermal properties of the fruit cake may have only a marginal effect on the overall heat transfer during the secondary phase. The reason for this is that the cake shrinks and, as a result, partially loses contact with the vial. This may further reduce the heat transfer and/or favor that of the vial itself, as already suggested by Yoon and Narsimhan [19].

**Figure 7:**
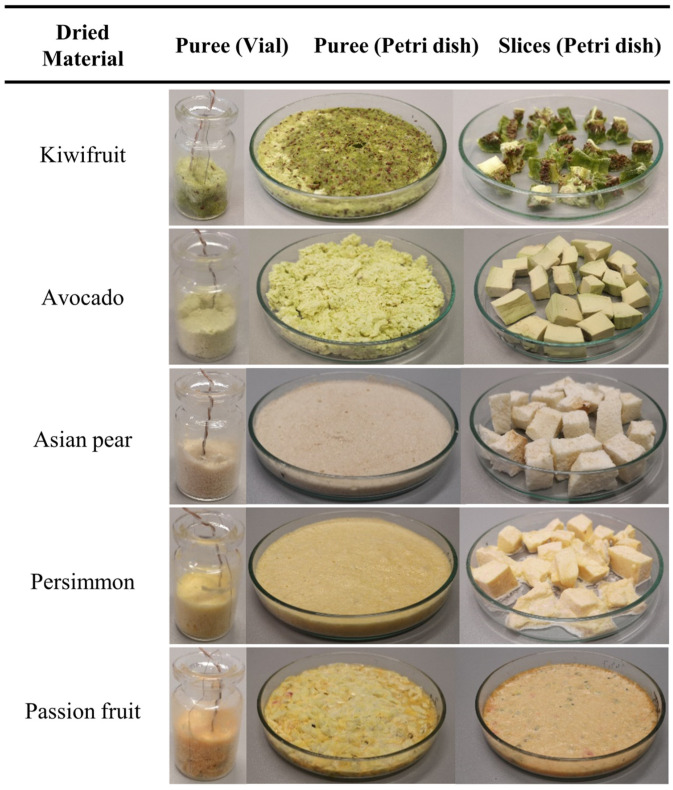
Fruit samples at the end of the freeze-drying cycle.

Finally, our measurements suggest that, although the amount of energy required for the desorption process is relatively small, the sensitivity of the heat flux sensor was sufficiently high. However, this is a critical moment as the final residual moisture content of the sample must be reduced to a recommended level of between 1% and 3% [21]. It would therefore be beneficial to utilize KF titration to determine the residual moisture change and compare the results with those obtained by the heat flux sensor. However, as this was not the main purpose of our study, it was outside the scope of the experimental measurements. The appearances of the fruit samples at the end of the freeze-drying cycle are shown in Figure 7.

### 3.5. Effect of Moisture Content on the Energy Flow

**Figure 8:**
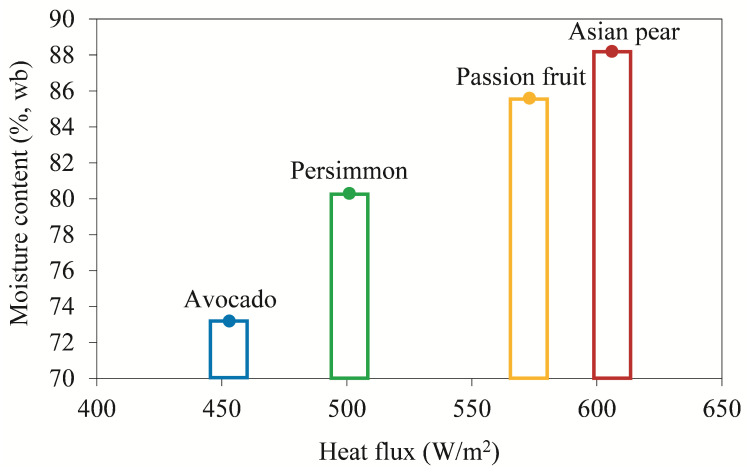
Effect of moisture content on the mean heat flux of different fruit samples.

After having characterized the transition from the primary drying phase to the secondary drying phase of the fruits, the effect of the moisture content on the energy flow was evaluated. Figure 8 shows the water content of the different fruit samples in relation to the mean heat flux measured during the 4 h of primary drying in a quasi-steady state. For the graphical representation of the dependence, moisture content values from Ref. [22] were used. The diagram shows the clear influence of water content on the magnitude of the heat flux, which is highest for the fruit with the highest water content. Conversely, the lowest heat flux is found in avocado, which has the lowest water content. From the above, it can be concluded that heat flux measurements can potentially be used as a tool to roughly estimate water content in samples of unknown composition.

## 4. Conclusions

The aim of the study was to evaluate the use of heat flux measurements for the noninvasive determination of the endpoint of the primary phase of lyophilized fruits (kiwifruit, avocado, Asian pear, persimmon, and passion fruit). Our experimental approach was based on a comparison between the evolution of the heat flux and temperature measurements during the lyophilization process. Using a thin-film heat flux sensor under the vial and a reference thermocouple placed inside the vial at its bottom, we observed both the random nucleation event and the transition from the primary drying phase to the secondary drying phase. The combination of both measurements proved to be one of the most reliable means of predicting the endpoint of the primary drying, overcoming the limitations of the existing studies that relied solely on invasive temperature measurements.

To assess the effect on the drying rate and primary drying time, different fruit species with different tissue compositions were analyzed. Several drying kinetics were obtained and analyzed from repeated measurements performed on specific fruit species in order to reduce the total drying time within a 24 h timeframe. Average heat fluxes in the primary drying phase ranged from 250 to 570 W/m^2^. In situ monitoring of the process parameters allowed optimization of the sublimation conditions, resulting in a significant change in the drying rate from a single drying iteration. For all drying runs, the reference temperature sensor in the vial showed excellent agreement with the response of the heat flux sensor during all three phases of the freeze-drying.

Further research is required to evaluate the optimum drying conditions for preserving the internal cell structure of different fruit species. Controlled nucleation would be beneficial to increase cake homogeneity. Our low-cost experimental approach is easily adaptable to any pilot-scale freeze dryer and, with appropriate sensor adaptation for sterile environments, could therefore be applied to industrial-scale freeze dryers.

## Figures and Tables

**Table 1:** Characteristics of the analyzed fruits.

Dried Material	Scientific Name	Sample Preparation/Mass		
		Puree (Vial)	Puree (Petri Dish)	Slices (Petri Dish)
Kiwifruit p0.14	*Actinidia deliciosa*	3.99 g	60.00 g	59.97 g
Kiwifruit p1.00	*Actinidia deliciosa*	4.02 g	59.91 g	59.93 g
Avocado	*Persea americana*	3.83 g	34.31 g	38.28 g
Asian pear	*Pyrus pyrifolia*	3.99 g	44.22 g	45.83 g
Persimmon	*Diospyros kaki*	4.41 g	55.10 g	42.72 g
Passion fruit	*Passiflora edulis*	5.12 g	34.31 g	39.12 g

**Table 2:** Freeze-drying protocols used.

Protocol No.		1	2	3
Dried Material		Kiwifruit p0.14	Kiwifruit p1.00	Avocado; Asian Pear; Persimmon; Passion Fruit
Freezing step	Shelf set temperature (°C)	−35	−35	−35
	Ramp rate (°C/h)	100	100	100
	Time (h)	4	3	3
Primary drying	Shelf set temperature (°C)	5	5	5
	Ramp rate (°C/h)	100	100	100
	Time (h)	12	12	16
	Chamber pressure (mbar)	0.14	1.00	0.14
Secondary drying	Shelf set temperature (°C)	30	30	30
	Ramp rate (°C/h)	100	100	100
	Time (h)	2	2	4
	Chamber pressure (mbar)	0.14	1.00	0.14

**Table 3:** Heat transfer coefficient *K_v_* as a function of chamber pressure.

Dried Material	Chamber Pressure (mbar)	Mean Heat Flux (W/m^2^)	Heat Transfer Coefficient, *Kv* (W/m^2^/°C)
Kiwifruit p0.14	0.14	197	9.8
Kiwifruit p1.00	1	737	42.7

## Data Availability

Data are available from the corresponding author upon reasonable request.
